# Integrating Air Quality and Public Health Benefits in U.S. Decarbonization Strategies

**DOI:** 10.3389/fpubh.2020.563358

**Published:** 2020-11-19

**Authors:** Ciaran L. Gallagher, Tracey Holloway

**Affiliations:** ^1^Nelson Institute Center for Sustainability and the Global Environment, University of Wisconsin—Madison, Madison, WI, United States; ^2^Department of Atmospheric and Oceanic Sciences, University of Wisconsin—Madison, Madison, WI, United States

**Keywords:** interdisciplinary, integrated assessment modeling, climate mitigation, electric vehicles (EV), renewable energy

## Abstract

Research on air quality and human health “co-benefits” from climate mitigation strategies represents a growing area of policy-relevant scholarship. Compared to other aspects of climate and energy policy evaluation, however, there are still relatively few of these co-benefits analyses. This sparsity reflects a historical disconnect between research quantifying energy and climate, and research dealing with air quality and health. The air quality co-benefits of climate, clean energy, and transportation electrification policies are typically assessed with models spanning social, physical, chemical, and biological systems. This review article summarizes studies to date and presents methods used for these interdisciplinary analyses. Studies in the peer-reviewed literature (*n* = 26) have evaluated carbon pricing, renewable portfolio standards, energy efficiency, renewable energy deployment, and clean transportation. A number of major findings have emerged from these studies: [1] decarbonization strategies can reduce air pollution disproportionally on the most polluted days; [2] renewable energy deployment and climate policies offer the highest health and economic benefits in regions with greater reliance on coal generation; [3] monetized air quality health co-benefits can offset costs of climate policy implementation; [4] monetized co-benefits typically exceed the levelized cost of electricity (LCOE) of renewable energies; [5] Electric vehicle (EV) adoption generally improves air quality on peak pollution days, but can result in ozone dis-benefits in urban centers due to the titration of ozone with nitrogen oxides. Drawing from these published studies, we review the state of knowledge on climate co-benefits to air quality and health, identifying opportunities for policy action and further research.

## Introduction

Fossil fuel combustion releases a complex mixture of emissions into the atmosphere, including carbon dioxide (CO_2_), carbon monoxide (CO), and nitrogen oxides (NOx = NO_2_ + NO), as well as primary particulate matter (PM), sulfur dioxide (SO_2_) and other compounds depending on the fuel and combustion process. Carbon dioxide emissions are primarily a concern due to their radiative impacts on the planet and role in climate change, while the other emissions impact human health through degraded air quality (1). In fact, exposure to health-damaging air pollution is the largest environmental impact on human health, responsible for 4.2 million lives lost each year (2).

Greenhouse gases (GHGs), especially CO_2_, are co-emitted with health-damaging air pollutants in power plants, vehicles, and other sources of fuel combustion. While GHGs themselves can have direct and indirect health impacts (3), they differ from “traditional air pollutants” with well-established effects on human health through respiratory exposure. Epidemiological and toxicological studies clearly identify adverse health outcomes associated with PM and certain reactive gases, including ozone (O_3_), CO, NO_2_, and SO_2_. Based on these health studies, these health-relevant pollutants have been regulated in the United States since 1970. The World Health Organization (WHO) also provides exposure guidelines for these traditional air pollutants (4). To clarify this distinction, we use the term “air quality” to refer to the abundance of chemicals in the air with well-established impacts on health, and “climate pollutants” to refer to chemicals in the air that affect the radiative balance of the planet by absorbing, reflecting, and/or re-emitting radiation. This distinction is important to emphasize “co-benefits” where actions to decrease climate pollution also benefit air quality and human health.

**Figure 1:**
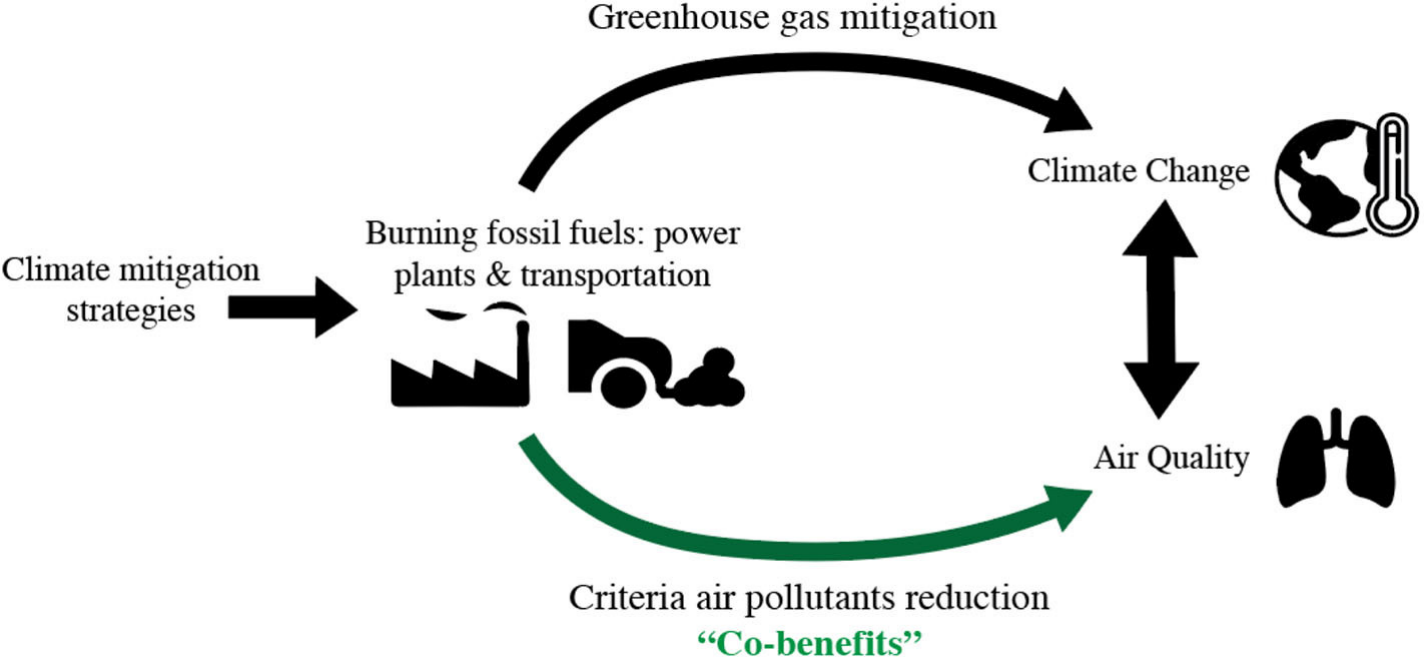
A schematic of the co-benefits from climate mitigation strategies due to the relationships between climate change and air quality.

The potential for energy system changes to offer co-benefits for both climate and public health is shown in Figure 1, where reductions in fossil fuel burning reduce a range of pollutants. The term “criteria” air pollutants refers to the six air pollutants regulated in the United States under the National Ambient Air Quality Standards: SO_2_, NO_2_, CO, PM, O_3_, and lead (Pb) (5). This group of pollutants overlaps with air pollution guidance from the WHO and threshold-based regulation in the European Union (6) and China (7). While all criteria air pollutants have well-established health impacts, PM and O_3_ have had a greater focus in the energy co-benefits literature due to their more frequent non-attainment status (8).

Both PM and O_3_ are considered regional air pollutants, mixing over synoptic scales due to chemical processes that promote their formation. Both PM and O_3_ are also sensitive to weather conditions, which affect mixing in space and chemical processes. These weather dependencies suggest that O_3_, and to a lesser degree PM, will be worsened by climate change, as discussed in the **Appendix** [e.g., (9, 10)]. The regional nature of these pollutants complicates the attribution of co-benefits. Local actions, such as retiring a coal-fired power plant, typically yield health and air quality co-benefits to the location where the emission reduction occurred (e.g., the county containing the retired power plant) as well as an area downwind. The location and magnitude of these benefits depend on weather patterns, atmospheric chemistry, and the distribution of the exposed population.

Evaluation of air quality and public health co-benefits of carbon strategies dates to the 1990s (11–13). Some of the earliest investigations of air quality co-benefits, then called “ancillary benefits,” were conducted by or for the U.S. Environment Protection Agency (14, 15). The earliest published studies utilized economic models that incorporated simplified air pollution scaling factors (16–18). These first studies demonstrated that there can be significant air quality co-benefits for climate and energy policies. For example, Tollefsen et al. (18) concluded that incorporating the climate impacts of air pollutants into European Union air quality strategies can avoid 7.3% of damages to human health and crops through efficiency gains. Bollen et al. (17) found that climate policies result in lower local air pollution and that the benefits largely outweigh the CO_2_ and PM_10_ emissions reduction costs.

Early studies were influenced by environmental concerns of acidification and eutrophication, which were reflected in international policies of the time including the 1979 Convention on Long-Range Transboundary Air Pollution (LRTAP) (19). One of the first reduced-form models used to evaluate co-benefits was the RAINS model (20), which optimized emission scenarios to co-manage acidification, eutrophication, and ground-level O_3_ impacts (17, 21, 22). In addition to RAINS, the Tracking and Analysis Framework (23), and stack-height-differentiated dispersion models (24) represented pioneering efforts to evaluate health and air quality impacts of energy system change. As efforts advanced to quantify benefits over multiple pollutants and multiple areas of impact, especially climate, this research domain began to be referred to as “co-benefits.”

With the evolution of computer processing, more advanced models could be developed and applied to air quality assessment. These “full physics” atmospheric models directly solve the mathematical equations representing fluid advection and atmospheric chemistry, from the regional to global scale. From the models, near-surface air quality was calculated as a function of changing emissions (25–27). These early studies supported the integrated, interdisciplinary analyses of air quality co-benefits that are the focus of this review.

In the 2000s, editorial essays and review articles identified the need for increased linkages among disciplines to quantify air pollution co-benefits (28–31). These papers argued that air quality analyses should be considered in climate change adaptation, planning, and policy decisions due to the public health potential of decarbonization measures. In particular, Bell et al. (28) reported a broad consensus across the literature that decarbonization can substantially improve air quality, despite the fact that publications to date had mostly conducted a single step of analysis (e.g., economic, emissions, air quality, or health), rather than providing an integrated assessment. A meta-analysis by Nemet et al. (32) found air quality co-benefits ($/tCO_2_) in the range of $2-196/tCO_2_. Studies included in that review included stack-height-differentiated dispersion models, simplified source-receptor relationship models, and scaling factors. Nemet et al. (32) did not include studies investigating the spatial and temporal air quality effects of clean transportation options, like electric vehicles (EVs), which were only beginning to emerge as a topic of analysis at the time. Analysis methods have advanced considerably since these earlier reviews, and we focus on the common findings and tradeoffs of newer analysis approaches.

Air quality models and integrated analysis methodologies enable more comprehensive as well as more spatially and temporally complex evaluation of air quality co-benefits. We focus on these model-based analyses of climate mitigation policies and technologies, as well as specific transportation and electricity sector decarbonization strategies. Across published studies, a number of consistent findings have emerged, as well as opportunities for policy action and further research. Section Study selection defines the criteria for study selection; Section Summary of published studies outlines the studies included in this review; Section Models and Methods presents the models and methods used across these co-benefit studies; Section Results presents results of our review; and section Conclusion presents conclusions and research needs. It should be noted that even the most advanced models do not determine policy outcomes. Energy planning takes place in a complex political and economic environment, both in the U.S. and in countries around the world. Even as models advance different actors will perceive results, including the distribution of benefits and costs, based on personal and institutional perspectives. Here we focus on the analysis tools available to support quantifying air quality and health co-benefits, recognizing that policy outcomes occur beyond the calculations of even the most sophisticated models.

## Study Selection

To conduct this review, we focus on peer-reviewed analysis included in the Clarivate Analytics Web of Science platform (WoS), as of in February 2020. The scope is limited to interdisciplinary analysis of changes in emissions and air quality from climate mitigation scenarios. We define climate mitigation to be a purposeful action taken to decrease CO_2_ emissions in order to slow global warming. These scenarios can include energy sector regulations, carbon taxes, or an increase in renewable energy or EVs. For inclusion in this review, we require that air quality analysis include the calculation of ambient concentrations from pollutant emissions; research studies that only used scaling factors to approximate public health benefits from emissions were not included.

Research on the health impacts of climate and air quality represents a small fraction of the work evaluating health impacts of climate alone or of air quality alone. Where a WoS search with keywords “climate” + “health” yields 28,580 results and “air pollution” + “health” yields 24,893 studies, a WoS search with the keywords “climate co-benefits” yields only 1,069 research studies and the keywords “air pollution” + “climate change” returns 3,540 results. The subset of co-benefits research appears to be 4–14% of the research on the health impacts of climate or air pollution alone. To systematically narrow the field of research to the interdisciplinary modeling studies at the heart of this review, we began with WoS keywords: “climate change air pollution co-benefit,” (200+ results) and “transportation air pollution co-benefit,” (50+ results).

Abstracts of these papers were reviewed to assess if the study: [1] quantified air quality co-benefits from climate mitigation actions and [2] analyzed both emissions and air quality. Papers were excluded that did not match these criteria. There are a number of closely related fields of study that we exclude from this review scope, including research that: [1] evaluates the climate forcing potential of fuel switching (33, 34); [2] omits an explicit calculation of emissions associated with the policy or technology (35, 36); [3] calculates (25, 37) or applies (11, 12, 17, 38, 39) scaling factors to approximate air quality impacts; [4] uses optimization methods to evaluate the most cost-effective way to achieve air quality and greenhouse gas reduction targets (11, 17, 40–43); [5] assumes a fixed criteria air pollutant or greenhouse gas emissions reduction (44–48), including zeroing out the emissions of a particular sector (49) and global warming temperature targets (50–55); [6] evaluates climate change's impact on air pollution without the context of a climate mitigation action (56, 57); [7] conducts life cycle assessments (58); and [8] has not been published in peer-reviewed journals (15, 59). Beyond the papers from the WoS keyword searches, we included research cited by retrieved papers or that cited retrieved papers, as long as papers met our inclusion criteria. We focus on United States policies and analysis; studies of other regions are included in Table S1 and discussed in Appendix II (27, 60–70).

Twenty-six studies met our inclusion criteria, which are listed in Tables 1–3. Seventeen of these quantify the human health and associated economic benefits of the air quality changes. The other nine report changes in ambient air pollution concentrations, but not health impacts. A related review on the air quality benefits of EVs was presented by Requia et al. (95), and included 65 studies, ten of which are also included here. That review includes research excluded from this review, such as life-cycle assessments and emissions-only analysis. By evaluating carbon, transportation, and electricity strategies, we focus here on consistent findings about fossil fuel reduction impacts on air quality and health.

**Table 1:** Scope and methods for research included in the literature review of climate policy air quality co-benefits.

**Authors**	**Study Purpose**	**Scale**	**Emissions model type**	**Air chemistry model type**
Burtraw et al. (23)	Quantify the ancillary benefits of a $25 and $75 carbon tax	National	Capacity expansion model	Reduced form
Zapata et al. (71)	Evaluate PM_2.5_ reduction co-benefits from California's AB 32	State	Emissions inventory	Full physics
Thompson et al. (72)	Compare national carbon reduction policies: Clean energy standard, Transportation policy, and Cap-and-Trade	National	Computable general equilibrium model	Full physics
Driscoll et al. (73)	Compare U.S. power plant carbon standards (three scenarios) and Carbon Price	National	Capacity expansion model	Full physics
Thompson et al. (74)	Compare subnational climate policies in U.S. Northeast: Cap-and-Trade vs. Clean Energy Standard	Regional	Computable general equilibrium model	Full physics
Barbose et al. (75)	Evaluate the co-benefits of complying Renewable Portfolio Standards as of 2013	National	Data and analysis tool	Reduced form
Ebrahimi et al. (76)	Evaluate air quality impacts of widespread electrification in California	State	Capacity expansion model	Full physics
Dimanchev et al. (77)	Compare subnational climate policies in the Rust Belt: Renewable Portfolio Standards (three scenarios) vs. Carbon price	Regional	Computable general equilibrium model	Reduced form
Zhao et al. (78)	Compare abatement costs and health co-benefits between two deep decarbonization scenarios in California	State	Emission inventory	Full physics

**Table 2:** Scope and methods of electricity generation and renewable energy deployment co-benefits research.

**Authors**	**Study Purpose**	**Scale**	**Emissions model type**	**Air chemistry model type**
McCubbin and Sovacool (79)	Evaluate the air quality benefits from deploying wind power in California and Idaho	Regional	Emissions inventory	Reduced form
Plachinski et al. (80)	Current, expected, proposed EE/RE state WI policies	State	Capacity expansion model	Full physics
Buonocore et al. (81)	Compare four scenarios: 500 MW wind, 500 MW solar, 500 MW reduced peak load, and 150 MW reduced baseload in six locations in the PJM Interconnection	Regional	Production cost model	Reduced form
Wiser et al. (82)	Evaluate benefits of solar PV deployment of 14% in 2030 and 27% in 2050	National	Capacity expansion model	Reduced form
Millstein et al. (83)	Quantify co-benefits from actual 2007–2015 PV and wind deployment	National	Data and analysis tool	Reduced form
Abel et al. (84)	17% electricity generation replaced with PV in Eastern U.S.	Regional	Production cost model	Full physics
Abel et al. (85)	12% summertime baseload electricity demand reduction stemming from energy efficiency measures	National	Data and analysis tool	Full physics
Buonocore et al. (86)	Scenarios of deploying 100–3,000 MW renewable energy (wind, utility solar PV, rooftop solar PV) in different U.S. regions	National	Data and analysis tool	Reduced form

**Table 3:** Scope and methods for transportation focused research.

**Authors**	**Study Purpose**	**Scale**	**Emissions model type**	**Air chemistry model type**
Thompson et al. (26)	Replace 20% of conventional vehicles with plug-in hybrid EVs during an August 2002 air pollution episode in the PJM area	Regional	Emissions inventory	Full physics
Brinkman et al. (87)	Ozone impacts of 30 vs. 100% plug-in hybrid EVs in Denver with and without controlled charging	Metropolitan	Electricity dispatch model, Emissions inventory, Mobile emissions simulator	Full physics
Grabow et al. (88)	Eliminate short automobile trips (<8 km) in 11 metropolitan areas in the Upper Midwest	Regional	Emissions inventory	Full physics
Bickford et al. (89)	Shift freight transport from truck to rail in the Upper Midwest	Regional	Emissions inventory, Mobile emissions database	Full physics
Weis et al. (90)	Life cycle analysis of the air quality impacts of BEVs, PHEVs, vs. conventional vehicles in the PJM Interconnection	Regional	Electricity dispatch model, Emissions inventory, Mobile emissions simulator	Reduced form
Razeghi et al. (91)	Comparisons of 40% PHEV vs. BEV penetration baseline vs. increased wind energy, and controlled charging or not scenarios in California's South Coast Air Basin	Regional	Electricity dispatch model, Emissions inventory, Mobile emissions database	Full physics
U.S. Environmental Protection Agency (92)	Co-benefits assessment of federal GHG emissions and fuel efficiency standards for medium- and heavy-duty vehicles	National	Emissions inventory	Full physics
Nopmongcol et al. (93)	Air quality impacts of electrifying 17% light duty and 8% heavy duty VMT as well as 79% off-road equipment	National	Optimization tool, Mobile emissions simulator, Mobile emissions database	Full physics
Pan et al. (94)	Comparison of moderate, aggressive, and complete EV scenarios in the Greater Houston Area	Metropolitan	Emissions inventory, Mobile emissions simulator	Full physics

## Summary of Published Studies

**Figure 2:**
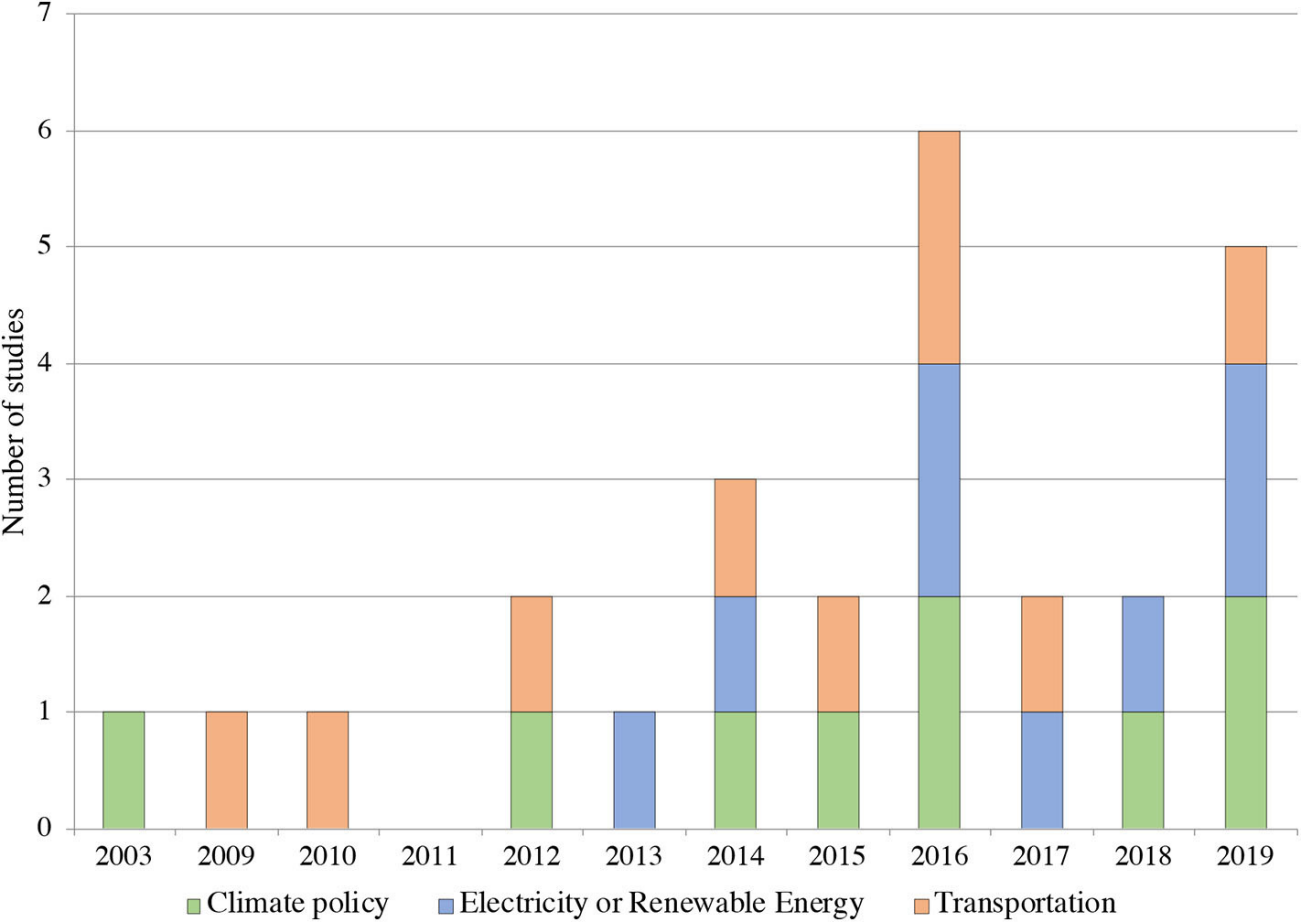
Histogram of research included in literature review by publishing date. The table is color coded to specify the type of climate mitigation strategy or policy evaluated in the studies: green indicates climate policies, blue indicates electricity generation or renewable energy deployment or policy, and orange indicates transportation. A majority of the research included in this review was published in the last 5 years, with the oldest study dating 2003.

The number of studies calculating air quality benefits of climate and energy in the United States has grown over the past 20 years, as shown in Figure 2, which includes all studies listed in Tables 1–3. In Table 2, green denotes climate policies, orange denotes transportation policies/technologies and blue denotes electric sector or renewable energy policies/technologies. The earliest research included here dates to 2003, with integrated air quality co-benefits research gaining momentum starting in the early 2010s. In the last 6 years, published research in the field has doubled or tripled, peaking at six studies in 2016 and five in 2019.

Studies focused on climate policies are listed in Table 1, which include carbon taxes, renewable portfolio standards, and other traditional policies leveraged by national and state governments to reduce carbon emissions. Analyses of multiple energy scenarios, including transportation and/or renewable energy strategies, have been categorized as a climate policy as they evaluate multiple strategies to decrease carbon emissions. All except Ebrahimi et al. (76) calculate health and associated economic benefits of climate policies. Most of these studies take an economic modeling approach to emissions calculations, with three using a computable general equilibrium model (72, 74, 77); three, a capacity expansion model (23, 73, 76); one, a data and analysis tool (75); and one, an emissions inventory (71). Most use a full-physics air quality model; only Dimanchev et al. (77) uses the reduced form model, InMAP.

Studies that evaluate changes to electricity generation, including reduced demand from energy efficiency as well as renewable energies, are listed in Table 2. All except Plachinski et al. (80) calculate the public health benefit and associated economic metrics of the changes to air quality. Four compare the co-benefits of different renewable energy deployments in various regions of the U.S. (79, 81, 83, 86); two, the air quality co-benefits of renewable energy deployed across the continental United States (82, 85). Emissions were quantified with data and analysis tools (83, 85, 86), a capacity expansion model (80, 82), a production cost model (81, 84) or an emissions inventory (79). Most use a reduced-form air quality model; only Abel et al. (84) and Plachinski et al. (80) use a full physics air quality model to evaluate energy efficiency and renewable energy benefits.

Table 3 lists transportation-focused research, which includes co-benefits analysis of mode shifting (88, 89, 93) and EV adoption (26, 87, 90, 91, 94). Three studies included the air quality impacts of EV adoption with a cleaner future electricity grid (87, 90, 91). Two studies investigated EV adoption in the Pennsylvania-New Jersey-Maryland (PJM) Interconnection: the regional transmission that serves parts of the Mid-Atlantic and Upper Midwest and is the most coal intensive grid in the United States (26, 90). Transportation-focused studies include two that focus on the air quality of metropolitan areas (87, 94) and one on a regional air basin (91). The smaller domains facilitate finer-scale analysis of spatial and temporal changes to O_3_ and other pollutants. As such, these studies primarily utilize full physics models [used in seven of the eight studies: (26, 87–89, 91, 93, 94)]. Quantification of health and economic benefits was rarer for the transportation studies; only two out of the eight included this analysis step (88, 94). Every study in Table 3 used an inventory approach to calculate vehicles tailpipe and/or EV charging electricity emissions. Mobile emissions inventories were supplemented with emissions factor databases (89, 91, 93). Mobile emissions simulators, like the EPA's MOVES model (89, 93, 94) and Argonne National Laboratory's GREET model (90), were also utilized to estimate mobile emissions. Emissions from charging EVs were estimated with a variety of methods including a unit commitment and dispatch model (87, 90), an electricity dispatch model (91), and an optimization tool (93).

## Models and Methods

As identified in our literature review (presented in Tables 1–3), research is expanding in the use of multi-model analysis to calculate air quality and health benefits of energy system change. Electricity dispatch models, economic models, and/or mobile emissions inventories are used to calculate time-sensitive and location-specific emission perturbations associated with a policy or technology change. The resulting emissions inventories are input into atmospheric chemistry models, or reduced-form models, to evaluate the impact of weather and chemistry on the distribution of ambient pollutants. Finally, the human health and economic impact from resulting ambient air quality concentrations are quantified using concentration response functions, population distribution data, and assumptions on the value of a statistical life. Table 4 introduces the models used in these studies.

**Table 4:** Table of models used in literature included in review.

**Model name**	**Acronym**	**Model Type**	**Developer**	**Studies used**
U.S. Regional Energy Policy	USREP	Computable general equilibrium model	MIT	(72, 74, 77)
Haiku	Haiku	Capacity expansion model	Resources for the Future	(23)
MyPower	MyPower		Meier engineering research	(80)
MARKet ALlocation	MARKAL		International energy agency	(76)
Regional energy deployment system	ReEDS		NREL	(82)
Integrated planning model	IPM		ICF	(73)
GridView	GridView	Production cost model	ABB	(84)
AVoided emissions and geneRation Tool	AVERT	Data and analysis tool	U.S. EPA	(83–86)
Greenhouse gases, regulated emissions, and energy use in transportation	GREET	Mobile emissions simulator	Argonne national laboratory	(90)
MOtor vehicle emission simulator	MOVES		U.S. EPA	(89, 93, 94)
Community multiscale air quality	CMAQ	Full physics air quality model	U.S. EPA	(73, 76, 80, 84, 85, 88, 89, 91, 92)
Comprehensive air quality model with extensions	CAMx		Ramboll environ	(26, 72, 74, 87, 93)
Weather Research and Forecasting coupled with Chemistry	WRF-Chem		NCAR	(78)
Tracking and analysis framework	TAF	Reduced form air quality model	U.S. DOE	(23)
Air pollution emission experiments and policy analysis	APEEP/AP2		N. Muller, Carnegie Mellon	(82, 83, 90)
CO–benefits risk assessment	COBRA		U.S. EPA	(75, 79)
Electrical policy simulation tool for electrical grid intervention	EPSTEIN		J. Buonocore et al. Harvard	(81, 86)
Estimating air pollution social impact using regression	EASIUR		J. Heo and P. Adams, Carnegie Mellon	(83, 86, 90)
Intervention model for air pollution	InMAP		C. Tessum, J. Hill, and J. Marshall	(77)
Environmental benefits mapping and analysis program	BenMAP	Health benefits model	U.S. EPA	(72–74, 78, 84, 85)

*Table includes type, developer, and studies used*.

Models used to quantify perturbations in emissions are shaded gray in Table 4. These include computable general equilibrium models (CGE) and power sector models, as well as transportation emissions models and evaluation tools developed by the U.S. EPA. Within power sector models there are multiple approaches (96, 97), two of which were used in the literature in this review: capacity expansion models and production cost models.

Here we discuss the emissions models in Table 4 as they relate to these general model categories:

- Economic CGE models simulate how different sectors of the economy impact energy and resource uses.
  - U.S. Regional Energy Policy (USREP) model (98) was used in three studies (72, 74, 77).
- Capacity expansion models, a subset of power sector models, simulate generation and transmission capacity, and find the optimal mix of generators to meet demand.
  - Haiku (99) was used in Burtraw et al. (23).
  - MyPower (100) was used in Plachinski et al. (80); this model has since been renamed JuiceBox.
  - International Energy Agency's MARKet ALlocation model (MARKAL) (101) was used in Ebrahimi et al. (76).
  - NREL's ReEDS model (102) was used in Wiser et al. (82).
  - ICF Resources L.L.C.'s Integrated Planning Model (IPM®) (103) was used in Driscoll et al. (73).
- Power sector models, a subset of production cost models, simulate chronological unit commitment and dispatch at high temporal resolution (minutes to hours) with detailed representation of transmission linkages.
  - ABB's GridView (104) was used in Abel et al. (84).
- A simplified data and analysis tool was developed by the U.S. EPA for wider adoption and easier implementation.
  - U.S. EPA's AVoided Emissions and geneRation Tool (AVERT) (105) was the most used emissions model, used in four studies (83–86).
- Transportation emissions depend on fleet vehicle types, fuel efficiencies and travel patterns, typically measured as vehicle miles traveled (VMT), which are in turn dependent on public transportation services, housing density, and job center locations (106, 107).
  - U.S. EPA's MOtor Vehicle Emission Simulator (MOVES) (108) was used in three studies (87, 93, 94) to simulate end-of-pipe emissions.
  - Greenhouse Gases, Regulated Emissions, and Energy Use in Transportation (GREET) model (109) was used in Weis et al. (90) to capture the entire fuel-cycle, including electricity emissions relevant for EVs.

Beyond the six studies using MOVES or GREET, twelve studies use bottom-up emissions inventories (89, 91, 93, 110). Emissions inventories and electricity dispatch models were used to allocate power plant emissions resulting from the additional vehicle charging load (87, 90, 91).

Air quality models are shaded in blue on Table 4. Two approaches have been used to extend emissions impacts to air quality and health: full physics models and reduced-form models. We use the term “full physics” to reflect the category of three-dimensional, chemical transport models (also called Eulerian models) which quantify changes in air quality due to weather, chemical processes, and deposition as well as emissions from natural and anthropogenic sources. In contrast, reduced form (or receptor-based) models are based on atmospheric model simulations and use simplified relationships (like source-receptor matrices) to estimate air quality and/or health associated with perturbed emissions. A third category of air quality model (Lagrangian dispersion models) is useful for source attribution and plume-modeling that is common for assessing the impact of individual point sources like power plants. These studies are outside of the scope of this review.

Three full physics atmospheric models were used across nineteen studies to evaluate spatial and temporal changes in ambient air pollution:

- U.S. EPA's Community Multiscale Air Quality model [CMAQ; (111)] was the most frequently used full physics model in these studies (73, 76, 80, 84, 85, 88, 89, 91). CMAQ is an open-source, state-of-the-art model that may be adapted for different spatial scales and global regions to calculate changes in ambient air pollution.
- Comprehensive Air Quality Model with Extensions [CAMx; (112)] is similar to CMAQ, but with somewhat reduced flexibility and associated computational cost. CAMx was used in six studies (26, 72, 74, 78, 87, 93).
- Weather Research and Forecasting model coupled with Chemistry [WRF-Chem; (113)] is similar to CMAQ and CAMx, but also allows for the analysis of air pollution impacts on weather and climate (e.g., the role of PM in cloud formation); WRF-Chem was used by Zhao et al. (78).

Seven reduced-form models were used across eleven studies to calculate ambient air quality effects, as well as health and economic metrics. Reduced-form models are computationally less intensive, allowing for the analysis of multiple emission scenarios, comparison across geographic regions, or the comparison of co-benefits across models. Three studies used multiple reduced-form models and presented health and economic co-benefits comparisons and averages across the models (83, 86, 90).

- Tracking and Analysis Framework [TAF; (114)] was used by Burtraw et al. (23).
- Air Pollution Emission Experiments and Policy [APEEP/AP2; (115)] was used in three studies (82, 83, 90).
- CO-Benefits Risk Assessment [COBRA; (116)] was used in two studies (75, 79, 83).
- Electrical Policy Simulation Tool for Electrical Grid Interventions [EPSTEIN; (117)] was used in two studies (81, 86).
- Estimating Air pollution Social Impact Using Regression [EASIUR; (118)] was used in three studies (73, 83, 90).
- Intervention Model for Air Pollution [InMAP; (119)] was used in Dimanchev et al. (77).

Reported human health benefit metrics include premature mortality, years of potential life lost, and disability-adjusted life years, which are calculated with concentration-response functions developed by epidemiology or toxicology health studies. Economic valuations of health outcomes are estimated with willingness to pay and value of a statistical life methods. EPA's Environmental Benefits Mapping and Analysis Program (BenMAP) (120) is the only model that exclusively calculates health and economic benefits. Eight studies linked full physics air quality model outputs with BenMAP to calculate premature mortality and the associated monetary benefit (72–74, 78, 84, 85, 88, 94). Three studies calculate public health benefits without the use of a model (60, 65, 71).

## Results

These 26 studies demonstrate the complexity of the air quality co-benefits research nexus. Some shared patterns and conclusions have emerged to further the scientific understanding of air quality co-benefits. Air quality co-benefits of decarbonization are not universal nor guaranteed (26, 74, 76), but do present large opportunities to decrease O_3_ and PM_2.5_ concentrations and reduce human population exposure to these health-damaging pollutants.

### Decreasing Fossil Fuel Use Reduces Emissions and Improves Air Quality

These studies consistently found that decreasing fossil fuel use resulted in a roughly linear decrease in precursor emissions, and a non-linear improvement in secondary air pollutant concentrations. The removal of fossil fuel emissions is generally proportional to avoided emissions of NO_x_, SO_2_, or primary PM_2.5_ (either on a sectoral or total basis). However, the response of ambient air pollution is sub-linear, showing percentage improvements much less than the percentage total reduction in precursor emissions.

Abel et al. (85) reported that a 12% summertime baseload electricity demand reduction due to energy efficiency could yield a decrease of 13.2% in NO_x_ emissions and a decrease of 12.6% in SO_2_ emissions. However, the model calculated ambient near-surface PM_2.5_ concentrations decreasing by 0.55% and O_3_ concentrations decreasing by 0.45%. The sub-linear relationship between emissions reductions and ambient concentrations may be explained by dispersion and mixing of emissions through the atmosphere, diluting the impact of near-surface concentration changes, as well as non-linearities associated with chemical processes and removal from the atmosphere through dry and wet deposition. This same pattern was found by Abel et al. (84) where a 17% solar photovoltaic (PV) scenario in the Eastern U.S. The 17% reduction in fossil-based grid-electricity reduced NO_x_ emissions by 20% and SO_2_ emissions by 15%, whereas ambient surface-level PM_2.5_ decreased by only 4.7%. Plachinski et al. (80) found that a 59% decrease in SO_2_ emissions produced a 3–13% decrease in ambient sulfate PM. Razeghi et al. (91) found that adding wind generation to charge EVs reduced ambient, near-surface PM_2.5_ and O_3_, but not to scale with the amount of renewable energy added. This sub-linear relationship between emissions reduction and air quality improvements highlights the complexity of air quality analysis and the value of advanced air quality models for policy assessments.

There is also interest in the impact of energy system changes on peak daily—or even hourly—ambient pollution levels. Air quality regulations for criteria pollutants in the United States are in part based on peak pollution days, through compliance with the National Ambient Air Quality Standards [NAAQS; (92)]. Air quality regulations are based on annual and short-term concentration averages, depending on the pollutant. Ozone is regulated based on maximum daily 8-h concentration averages; PM_2.5_, on both a yearly and daily standard (8). To evaluate whether energy system changes improve compliance with these air quality standards, assessments must evaluate whether air quality improvements occur on the “dirtiest” air pollution days.

The evaluation of regulatory outcomes in these studies builds on earlier co-benefit analyses conducted for federal agencies (14, 15). More recently Regulatory Impact Analyses (RIA) for carbon-related federal regulations have also addressed the impact on NAAQS attainment. For example, the Phase 2 GHG Emissions and Fuel Efficiency Standards RIA reported that nine counties modeled to exceed the NAAQS annual PM_2.5_ standard in the reference case would have decreased annual PM_2.5_ concentrations by 0.01–0.03 μg/m^3^ with the vehicle standards (92). Potential NAAQS benefits of energy system change were also included in some published studies, typically requiring the use of advanced air quality models like CMAQ.

The solar energy study by Abel et al. (84) found the largest reductions in PM_2.5_ on the most polluted days, suggesting that increased PV offers a strategy for potential compliance with the NAAQS for PM_2.5_. The energy efficiency study by Abel et al. (85) calculated a reduction in the number of days violating the O_3_ NAAQS in counties across the United States. An analysis of California's climate legislation by Zapata et al. (71) found PM_2.5_ concentrations were reduced by 2–3 μg/m^3^ over most urban areas during weather conditions that often produce air pollution exceeding federal standards (see **Appendix I** for supplementary information on how weather and climate change impact air quality). In the same study, the San Joaquin Valley had increased PM_2.5_ concentrations of 0.3 μg/m^3^ due to further use of dairy biogas as renewable energy. Overall, the literature suggests that decarbonization strategies impact on peak pollution days, suggesting energy system change as a potential strategy to support PM_2.5_ and O_3_ NAAQS attainment.

Just as Zapata et al. (71) found greater PM_2.5_ concentration reductions over urban areas, Zhao et al. (78) also reported uneven spatial distribution of air quality improvements from deep decarbonization scenarios in California. The largest PM_2.5_ reductions occurred in the largest four metropolitan regions, coinciding with some of the most polluted urban areas of the state. In that study, air quality improvements varied across the decarbonization scenarios.

### Reductions in PM_2.5_ Yield Maximum Health Benefits

Because PM_2.5_ is the air pollutant most directly linked to premature mortality (121), reducing fine PM results in the highest public health benefits. The U.S. EPA has determined a causal relationship exists between PM_2.5_ and mortality, for both long- and short-term exposure (122). The U.S. EPA economic analysis guidelines use the central estimate (mean) value of a statistical life (VSL) of $7.4 million ($2006) (123). A large population's exposure to even a small reduction in PM_2.5_ concentrations can decrease mortality and consequently result in substantial monetized co-benefits.

The majority of health benefits reported in the studies were due to the changes in PM_2.5_ levels, particularly sulfate PM_2.5_ from coal-fired power plants (26, 72, 73, 75, 82). In Driscoll et al. (73), the reduction of PM_2.5_ avoided about 3,000 premature deaths, ten times the number of premature deaths avoided by ambient O_3_ reductions. Wiser et al. (82) reported that reducing SO_2_ emissions, and subsequently particulate sulfate concentrations, accounted for more than 60% of the monetized co-benefits.

### Displaced Coal Generation and Population Exposure Strongly Affect Co-benefit Valuation

Renewable energy deployment and climate policies have the highest health and economic benefits in highly populated regions near or downwind from coal-powered electricity generation. Per energy units, coal power plants emit more CO_2_ than other energy sources, including natural gas (124), and have higher emissions of SO_2_, NO_x_, and PM_2.5_ (125).

Because coal has historically been cheaper than natural gas, many regions of the United States rely on coal-fired power plants for base-load electricity. However, the energy mixing is changing rapidly as natural gas has become abundant and cheap due to unconventional production (126). In addition, decreasing costs of wind, solar, and storage technologies have also made these alternatives cost-competitive (127). Because coal power plants are retiring each year, the choice of base year for health impact assessments can strongly affect calculated health benefits. In fact, a retrospective evaluation of 2013 U.S. renewable portfolio standards compliance obligations found that reductions in SO_2_ emissions from coal power plants accounted for 77–83% of calculated public health benefits (75).

In model-based studies, both Buonocore et al. (86) and Millstein et al. (83) found the largest health co-benefits from renewable energy and energy efficiency in the Upper Midwest and Mid-Atlantic. These regions have the most coal electricity generation in the United States. Both studies also found the smallest air quality co-benefits in California. California has one of the cleanest electricity generation mixes, with only one in-state coal-fired power plant supplying 0.14% of the state's electric load (128). Large regional differences in air quality benefits from solar and wind renewable energy deployment are attributable to the fuel displaced by renewable energies (83). Buonocore et al. (81) found that avoided SO_2_ emissions from coal displacement dominated total air quality benefits, due to their role in forming sulfate PM_2.5_. Abel et al. (84) also found the Ohio River Valley had the largest air quality benefits within their study region of the Eastern United States. When considering the potential for solar to reduce coal emissions, Wiser et al. (82) reported the greatest co-benefits in Texas, Oklahoma, Louisiana, Arkansas, and other parts of the Southeast due to a climate favorable for solar energy deployment.

The health benefits of air quality improvement depend on population exposure. As a result, co-benefits are greatest where large populations experience large air quality improvements (75, 78, 84, 86). These air quality and health outcomes occur at the urban and regional scale, providing local benefits to reduced fossil fuel use, complementing the global benefits of reducing GHG emissions. Furthermore, the air quality and health benefits occur immediately after fossil fuel emissions decrease, in contrast to the decadal to centuries timescales over which climate responds to changes in radiative forcing. In fact, Driscoll et al. (73) found that implementing national carbon standards for power plants yields immediate, local benefits. Buonocore et al. (86) found the highest co-benefits in the Upper Midwest and the lowest in California due both to displaced fossil fuel and population exposure. This localized aspect of air quality improvement has environmental justice implications. Since many disadvantaged communities are disproportionately likely to live next to pollution point sources (i.e., power plants and industry facilities), their exposure to pollution could be reduced to a greater degree (129).

### The Monetized Value of Co-benefits Typically Exceed Implementation Costs

**Figure 3:**
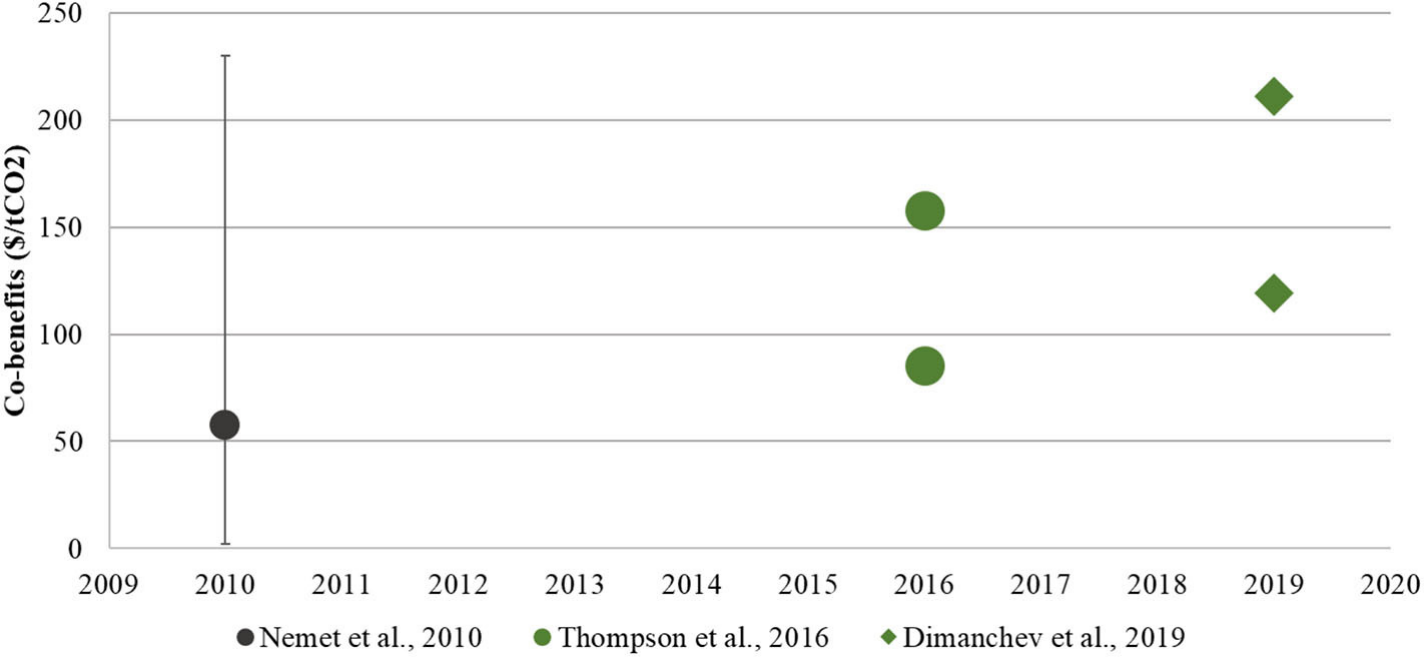
Graph of the three studies that include air quality and public health co-benefits monetized as U.S. dollars per ton of CO_2_ ($/tCO_2_). These are reported from left to right by date of study (2010–2019). Nemet et al. (32)'s range of co-benefits value review is included as a comparison. All values have been adjusted for inflation to 2019 US$ (36, 74, 77).

Monetized air quality health co-benefits were found to offset costs of climate policy implementation or renewable energy deployment, as shown in Figure 3. In comparing a national clean energy standard, an economy-wide cap and trade policy, and a clean transportation policy, Thompson et al. (72) calculated that 26–1,050% of the policy implementation costs would be offset by monetized health benefits. A comparison of regional climate policies found that the benefits of a cap-and-trade system exceed the implementation costs by more than a factor of 8, and the benefits of a clean energy standard exceed the implementation costs by almost a factor of 2 (74). Dimanchev et al. (77) estimate that co-benefits from subnational renewable portfolio standards outweigh implementation costs by 34%. A retrospective study of renewable portfolio standards reported that the yearly monetized public health co-benefits exceed yearly compliance costs of this climate policy (75). These U.S. focused studies correspond with the conclusions of Nemet et al. (32), which concluded that the economic co-benefit estimates had a similar order of magnitude to the policy abatement costs.

**Figure 4:**
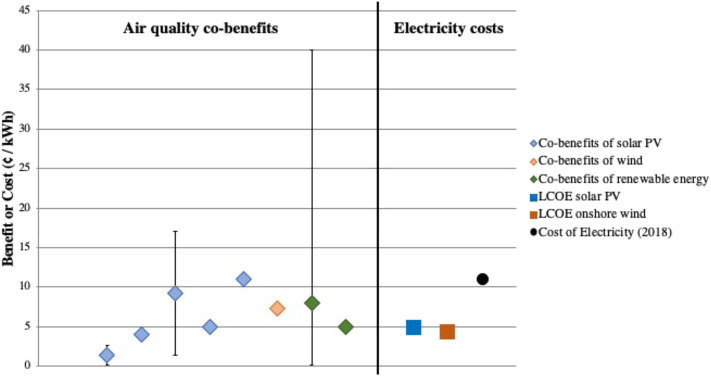
Graph of the five studies that include air quality and public health co-benefits monetized as cents per kWh (c/kWh) as well as levelized cost of energy (LCOE) for solar PV and onshore wind and U.S. average electricity costs in July 2018 (77, 81–83, 85).

Figure 4 compares monetized air quality benefits of PV and wind vs. deployment costs reported as $/MWh or c/kWh ($0.01/kWh). Millstein et al. (83) calculated the benefits to be equal to or greater than the levelized cost of electricity (LCOE) for wind and utility solar. LCOE describes the cost of electricity per electricity produced ($/MWh or $/kWh) and is a commonly used metric to compare different energy generation types. Buonocore et al. (81) also reported that the monetized economic benefits of renewable energies were the same order of magnitude of the U.S. Energy Information Administration's (EIA) LCOE for renewables. The one exception is residential solar PV, which has high deployment costs stemming from the lack of economies of scale. Dimanchev et al. (77)'s calculated benefits per kWh of renewable energy deployed (average of 8c per kWh) also exceed the solar and wind's LCOE of 4c per kWh. Wiser et al. (82) analyzed increased solar of 14% by 2030 and 27% by 2050, which reduced air pollutant emissions by about 10%. This resulted in co-benefits valued at 1.4c of co-benefits per kWh of solar installed. The levelized cost of electricity for wind and solar PV as well as the price of electricity are included as a comparison (85, 130). All but two co-benefits exceed the LCOE of renewables, indicating that air quality benefits alone exceed the cost of deployment.

The reported air quality co-benefits research almost universally demonstrates that both renewable energy deployment and climate policies are cost-effective ways to reduce the public health burden of energy, supporting the synergy between climate and air quality policies. Comparisons of different types of carbon policies concluded that more flexible carbon policies are less costly but can yield similar air quality co-benefits. The regional economy-wide cap and trade policy analyzed by Thompson et al. (74) was cheaper than the compared regional clean energy standard while the air quality and human health benefits for both policies were comparable. Thus, the benefits per implementation cost were higher for the cap-and-trade. Similarly, Thompson et al. (72) reported diminishing benefits with increased stringency of national carbon policies. Driscoll et al. (73) also found that stringent but flexible power plant carbon standards yielded the most health benefits, and Dimanchev et al. (77) found that a price on carbon yielded higher human health co-benefits compared to a renewable portfolio standard since the carbon standard applied to the whole economy and as such was more flexible. A study of deep decarbonization policies in California found the more expensive technological pathway (by $25 billion) still yielded a larger net benefit (health co-benefits minus GHG abatement costs) by $59 billion (78).

### Some Energy Strategies Yield a Mix of Benefits and Disbenefits

Air quality and health disbenefits can occur in some scenarios, depending on the implementation of an energy policy. These disbenefits have occurred in models that capture economic feedbacks from changing fuel prices, sectoral shifts in energy demand and/or non-linear chemical processes associated with O_3_ formation.

As an example of economic factors leading to isolated disbenefits, Thompson et al. (74) found that a Clean Energy Standard in the Northeast resulted in decreased demand for coal in that region, subsequently lowering coal prices. As a result, the demand for coal increased in other regions. Sectoral shifts in energy demand can move emissions, air pollution, and health costs from one area to another, causing localized increases in the region of increased fossil fuel combustion. Electrifying transportation is the most widely studied sectoral shift, as greater EV utilization reduces on-road combustion by conventional vehicles, but may increase power plant combustion to provide electricity to the EVs. This shift typically affects the location of emissions, with lower emissions on roadways and potentially higher emissions at power plants. The shift also affects the timing of emissions, with lower emissions during the day due to reduced on-road emissions during driving. Instead, power plants are operating to charge EVs, especially overnight. Thus, air pollution emissions shift spatially from the tailpipe to nearby power plants and temporally from daytime to nighttime (26, 87). Ebrahimi et al. (76) found that electrifying transportation systems in California resulted in air quality improvements in highly populated areas with poor air quality, but also localized O_3_ and PM_2.5_ concentrations next to electricity generating units. The potential disbenefits of vehicle electrification depend on the sources of electricity. Ambient PM_2.5_ has been found to increase if EVs charging is supplied by coal-power plants (26). However, a study in California did not see increased PM_2.5_ concentrations (91) because the electricity grid serving Los Angeles relies on no coal-fired electricity generation. While EVs can improve local air quality, benefits may be lost if charging is powered by coal power plants. Weis et al. (90) found that switching from conventional vehicles to EVs with the contemporary PJM grid resulted in worse health outcomes; EVs were found to produce health benefits if coal plants were retired and wind capacity increased (90). Another example of sectoral shifts yielding a mix of benefits and disbenefits was found by Bickford et al. (89), where a modal shift of freight movement from truck to rail reduced NO_x_, PM_2.5_, and O_3_ concentrations, but found local increases in pollution close to the railroads with higher levels of activity.

Potential disbenefits from emission reductions can also arise from the non-linear chemical processes controlling O_3_ formation. Chemical production of O_3_ is limited by the availability of VOCs (Volatile Organic Compounds) relative to NO_x_, so areas with high NO_x_ and lower VOCs (such as urban centers and power plant plumes) can exhibit a negative relationship between NO_x_ and O_3_. In these conditions, reducing NO_x_ actually increases O_3_ concentrations. Studies reviewed here found an increase in urban O_3_ in Houston, Denver, Newark, and a regional study of the PJM territory (26, 87, 94).

Despite this localized disbenefit, multiple studies found O_3_ concentrations to decrease with replacing conventional vehicles with EVs, especially on peak pollution days. The regional PJM from Thompson et al. (26) found 20% replacement with plug-in hybrid EVs resulted in O_3_ decreases between 2 and 6 ppb across the region's urban areas during a modeled historic air pollution episode. An analysis of 100% PHEVs penetration in Denver, Colorado found a 2–3 ppm reduction of O_3_ on days with the highest O_3_ concentrations in the base-case (87). In Houston, modeling 35% EV penetration resulted in an air pollution episode average O_3_ concentration decrease of 3–4 ppb (94). Modeling 40% light-duty electric vehicles penetration in the Los Angeles air basin, Razeghi et al. (91) found widespread O_3_ decreases across their study region, with localized O_3_ increases near electricity generation units. Grabow et al. (88) also saw widespread benefits and local O_3_ disbenefit when replacing short automobile trips (<8 km) in the Upper Midwest with active transit options, like walking and biking. Concentrations of PM_2.5_ decreased across the region, including cities (especially during high pollution episodes); O_3_ decreased in non-urban areas and small cities, but increased in larger cities.

## Conclusion

Coupled and reduced-form models reflecting energy system change, atmospheric processes, and health impacts draw from and require multidisciplinary thinking and teams. Studies using a variety of approaches consistently find a high value of air quality and health benefits to clean energy. This research has increased awareness that co-benefits are an important component of energy policy valuation. Across published studies, both reduced-form and advanced models are used, often with qualitatively similar results. This is consistent with the structured reduced-form and full-physics model comparison in Gilmore et al. (131). While reduced-form models provide policymakers a general estimate of co-benefits, they are not typically consistent with the rigorous expectations of compliance with U.S. Clean Air Act planning (such as State Implementation Plans).

Even given the differences in methods and assumptions, overall the air quality conclusions are consistent across research studies. Monetized public health benefits fall within a 50–250 $/tCO_2_, aligning with a previously reported range. The central values of air quality co-benefits (c/kWh) deviate little, with a standard deviation of only 3 c/kWh. These health valuations are dominated by the mortality impacts of PM_2.5_ exposure. It is unclear if a similar level of agreement would exist for specific health outcomes and/or pollutant exposure beyond PM_2.5_.

Although air quality and climate change have environmental justice consequences, none of the studies discussed here specifically consider low-income and communities of color. These communities have historically faced the greatest air pollution burdens (129) and are now the most vulnerable to climate change impacts (132). Tools exist to support this type of analysis, including explicit characterization of age, race, and ethnicity in BenMAP, and environmental justice characterization in the reduced-form InMAP model.

Another gap in published studies includes the potential “leakage” of emissions and air quality impacts from regional or statewide climate mitigation policies and strategies. Given the state-to-state transfer of electricity, as well as state-to-state transfer of air pollution in the atmosphere, any analysis over a fixed region will not capture the full impact of energy policies. Only one study quantified this leakage of indirect air quality increases from a northeast clean energy standard and carbon price (74). The leakage impacts of regional and statewide policies would be a valuable issue to explore further, given the interconnected nature of energy systems, transportation infrastructure, and federal air pollution control in the United States.

Decarbonization policies have effects beyond climate mitigation and air quality. Household energy budgets and lifestyle changes (e.g., increased physical activity) are also affected and have interlocking impacts on public health. For example, Grabow et al. (88) found eliminating short vehicle trips (≤8 km round trip) and replacing half of those trips with bicycling reduces premature mortality per year 2-fold: about 600 fewer deaths from improved air quality and almost 700 fewer deaths from increased physical activity. These additional impacts could also be integrated into health co-benefits analyses.

Studies to date showcase the potential for energy system change to benefit air quality and public health. However, it is still unusual for energy changes to be included in state-level planning under the Clean Air Act. Energy analyses regularly characterize CO_2_ emission impacts, but rarely NO_x_ or SO_2_. The disconnect between these issues misses the potential for “win-win” solutions for carbon reductions and public health benefits. Moving forward, policy and planning stand to benefit from the wealth of models and methods to support integrated analysis of energy, air quality, and health. The beneficial health findings can promote decarbonization strategies, and multi-pollutant solutions for health-damaging air pollution.

## Data Availability Statement

The original contributions presented in the study are included in the article/supplementary material, further inquiries can be directed to the corresponding author/s.

## Author Contributions

CG and TH contributed to the design and execution of the literature review, to the analysis of the results, and to the writing of the manuscript.

## Conflict of Interest

The authors declare that the research was conducted in the absence of any commercial or financial relationships that could be construed as a potential conflict of interest.
