# Supplementary material for: Integrating Air Quality and Public Health Benefits in U.S. Decarbonization Strategies
Source: Front Public Health. 2020 Nov 19;8:563358. doi: 10.3389/fpubh.2020.563358 (PMC7717953; doi:10.3389/fpubh.2020.563358)
Supplement: Supplementary file 1 [file Data_Sheet_1.docx]

***Supplementary Information***

**I: Weather and climate change effect on air quality**

Weather hugely impacts ambient air quality, which can add another layer of complexity to air quality analysis. Wind can transport air pollution away from sources, so it can dilute pollution in certain areas and concentrate pollution in others (Jacob 1999). Temperature inversions, where a layer of warm air traps in colder air, also impact pollution transport (Jacob 1999). Temperature inversions result in stable air, meaning little to no wind, and air pollution can build up. Temperature also affects reaction rates of air pollutants, most notably O_3_ (Cox and Chu 1996). Ozone formation is strongly correlated with temperature, so more O_3_ forms on warmer days.

Full-physics models calculate how emissions and weather interact in space and time to affect air quality. Using Eulerian air quality models in this research space has shown that the spatial distribution of benefits depends on wind patterns. Modelling air quality over Wisconsin, Plachinski et al. (2014) observed that wind and other atmospheric conditions of the Great Lakes region affected ambient concentrations. Abel et al. (2019) found that the largest pollution decreases occurred close to and downwind of coal power plants. Another analysis only considered the population exposure of air pollution downwind of power plants, under the assumption that this is where all produced pollutants were transported (Buonocore et al. 2016). An analysis of California’s AB 32 legislation found discrepancies in air quality outcomes across air basins when considering the whole state, since air basins have distinct wind patterns that can transport or trap air pollutants (Zapata, Muller, and Kleeman 2013). The San Joaquin Valley has unique meteorology and topography that trap wind and air pollution, which contributed to the air pollution increases found in that study.

Since weather has such a significant impact on air quality, consideration of how global climate change will modify weather patterns is another important factor when evaluating future air quality. Climate change and air quality influence each other in numerous, but sometimes opposing, ways (Fiore, Naik, and Leibensperger 2015). Atmospheric chemists are in conclusion that a warmer climate will lead to more O_3_ formation (Gonzalez-Abraham et al. 2015; Jacob and Winner 2009; Weaver et al. 2009). How climate change will affect PM_2.5_ concentrations, however, is less certain (Jacob and Winner 2009). Increased frequency and intensity of droughts and wildfires will likely increase PM_2.5_ emissions (Spracklen et al. 2009). Climate change’s impact on secondary PM_2.5_ depends on the type of fine particulate matter, as some species may have higher rates of formation in warmer temperatures (Jacob and Winner 2009). Additionally, the precipitation and ventilation sinks of PM_2.5_ will also be impacted by climate change, although by what magnitude is highly uncertain and will likely vary spatially (Jacob and Winner 2009). Furthermore, air pollutants can either contribute to or minimize global warming. Black carbon and O_3_ have positive radiative forcing, whereas other air pollutants, like sulfate particulate matter, can have a cooling effect on the climate (Unger 2012).

Global warming induced weather changes also exacerbate air quality through increased electricity demand. Hotter days, which will likely occur more often in the future, have higher electricity demand due to increased usage of air conditioning (Meier et al. 2017). Higher electricity demand increases energy sector emissions and worsens air quality (Abel et al., 2018b; Meier et al., 2017). These weather conditions also coincide with the weather that exacerbates air quality: hot, stagnant air. Although not included in this literature since they do not model a greenhouse gas emissions mitigation strategy, a vein of air quality co-benefits research has considered climate change’s impact on electricity demand and air quality by modeling mid-21^st^ century climate scenarios. Chen et al. (2015) predict the climatic warming will increase NO_x_ emissions in the Mid-Atlantic region, even though more stringent climate change and pollutant control policies will be implemented. This is expected to occur especially on warmer days, which is when O_3_ episodes are likely to occur. This study is a worst-case scenario, as no new renewable energy or closed power plants were included in the scenario, but it does demonstrate the complexities that other research studies ignore by modeling perturbed emissions across identical weather scenarios. Abel et al. (2018a) concluded that 3.8% of PM_2.5_ concentrations and 6.7% of O_3_ concentrations in 2050 would be contributable to the increased use of air conditioning in response to the higher temperatures.

**II: Non-U.S. based research**

Non-U.S. and global analyses are listed in Table S1, color coded to reflect the category of research. Six evaluate climate policies and the other six consider urban transportation climate mitigation strategies. Most focus on sub-regions of the world, with only two global analyses (McCollum et al., 2013). Five evaluate the air quality effects of EV adoption and/or other clean transportation policies in cities and countries in Europe and Asia (Soret, Guevara, and Baldasano 2014; Ferrero, Alessandrini, and Balanzino 2016; Li et al. 2016; Liang et al. 2019; Wolkinger et al. 2018). Similar to the U.S. based transportation analyses, these focus on limited geographic domains including single cities and regions. The three other international studies evaluate numerous policies and energy futures for Beijing, India, Eastern Africa, and worldwide (Zhao, Xu, and Hao 2011; McCollum et al. 2013; K. Chen et al. 2019; Van De Ven et al. 2019).

Some of the models used by the international research studies are also used in U.S. based research and thus were discussed in Section IV. These include GREET (Liang et al. 2019), MOVES (Lin et al. 2020), CMAQ (Zhao, Xu, and Hao 2011; Soret, Guevara, and Baldasano 2014; Li et al. 2016; K. Chen et al. 2019), and WRF-Chem (Peng et al. 2018). In addition, the following air quality models were used only in international/global research studies:

- Greenhouse Gas and Air Pollution Interactions and Synergies (GAINS) (Amann et al. 2009) is an international reduced-from air quality model that is an evolution of the earlier RAINS model. It is used by only one study (McCollum et al. 2013).
- TM5-Fast Scenario Screening tool (TM5-FASST) (Van Dingenen et al. 2018) is another reduced-from air quality source-receptor model and was used in one study (Vandyck et al. 2018).
- Graz Lagrangian Model (GRAL) is a lagrangian dispersion model, the only langrangian model used across all studies, and was utilized only by Wolkinger et al. (2018).

Much of the international research in this field has focused on India or China. Developing countries, like countries in Africa, India and until recently China, rely on solid fuels (e.g. wood, kerosene, charcoal) for heat and light. These fuels emit a lot of particulate matter and other health impacting air pollutants in indoor living spaces, making indoor smoke exposure from solid fuels the greatest global environmental health burden (Lelieveld et al. 2015). Electrification scenarios that include reducing traditional biomass burning drastically improve indoor air quality and health outcomes (Chen et al., 2019; Peng et al., 2018; Zhao et al., 2011). Chen et al. (2019) found reducing solid fuel burning most benefited air quality improvement across a variety of measures to reduce air pollution. Peng et al. (2018) reported that electrification in China resulted in improved air quality and public health benefits when coupled with decarbonization of the electricity sector, from 75% coal powered to 52%. In eastern Africa, energy technology subsidies result in more GHG emissions reductions than expansion of biomass resources and improvement in both household air pollution and ambient air quality (Van De Ven et al. 2019).

A subset of international health co-benefits research evaluates the human health co-benefits of the international Paris climate agreement (Sampedro et al. 2020; Vandyck et al. 2020; Markandya et al. 2018; Shindell, Lee, and Faluvegi 2016; Rauner, Hilaire, et al. 2020; Rauner, Bauer, et al. 2020; Vandyck et al. 2018). Most of these papers do not fit in our scope, since they evaluate the achievement of limiting warming by 2°C and not specific decarbonization measures. One paper in this research subset, however, does evaluate Nationally Determined Contributions (NDC) by individual countries (Vandyck et al. 2018). This study found air quality co-benefits for most pollutants and regions, but with some variability. For example, reducing the combustion of fossil fuels for energy directly reduced GHGs and criteria air pollutants, but other climate mitigation measures (i.e. biomass energy, biofuels, and land use change) had more complex air quality results. Overall, by 2030 the NDCs resulted in significant localized decreases in PM_2.5_ concentrations and a widespread decrease in the ozone mixing ratio. These air quality improvements led to 71 to 99 thousand avoided premature deaths globally in 2030, compared to the reference case of current climate policies.

One global study concluded that climate mitigation strategies can improve air quality more than current and planned pollution controls, because decarbonization can so greatly reduce PM_2.5_ concentrations (McCollum et al. 2013). Furthermore, pursuing only climate mitigation measures is less costly than pursuing climate and pollution controls simultaneously.

The non-U.S. focused transportation studies align with the conclusions of the transportation research based in the United States. For example, a study of vehicle electrification in China found larger health benefits in megacities, driven by the large population exposure reduction (Liang et al. 2019). This paper also found spatial shifts of air pollution from transportation corridors to areas nearby power plants. International research also found transportation electrification to increase O_3_ formation in urban centers and over freeways due to the reduction of O_3_ scavenging by NO_x_. Research in Taiwan found overall decreases in O_3_ concentrations except for a 2 ppb increase downtown metropolitan Taipei (Li et al. 2016) while a study in Spain also reported overall O_3_ decreases with a 2 ppb increase in Madrid and Barcelona downtown areas (Soret, Guevara, and Baldasano 2014). Overall O_3_ concentrations decreased on peak pollution days in the international studies as well. In Taiwan, 100% vehicle electrification would significantly reduce O_3_ air pollution episodes on average by 7 days per year and in the most polluted regions by 35 days per year (Li et al. 2016). In an Italian study, air quality improvements were particularly evident during air pollution episodes, even with the lowest EV penetration scenario of 25% (Ferrero, Alessandrini, and Balanzino 2016).

Similar to Grabow et al. (2012), a study that evaluated the co-benefits of urban transportation climate mitigation policies in three Austrian cities found increased physical activity reduced premature mortality more than the air quality improvement (Wolkinger et al., 2018).

| **Authors** | **Study Purpose** | **Scale** | **Emissions model type** | **Air chemistry model type** |
| --- | --- | --- | --- | --- |
| **Zhao et al., 2011** | Compare five future energy scenarios in Beijing | International Subnational | Emissions inventory | Full physics |
| **McCollum et al., 2013** | Several hundred alternate energy futures with different air pollution, energy security, and climate change policy prioritizations | Global | Integrated assessment model | Reduced form |
| **Soret et al., 2014** | Compare three vehicle electrification scenarios in Barcelona and Madrid | International Metropolitan | Mobile emissions simulator | Full physics |
| **Ferrero et al., 2016** | EVs impact on air quality near a highway in Milan, Italy | International Metropolitan | Emissions inventory | Full physics |
| **Li et al., 2016** | Replace 100% light duty conventional vehicles with EVs in Taiwan under different electricity generation scenarios | International National | Emissions inventory | Full physics |
| **Peng et al., 2018** | Evaluate the effects of electrification and moderate decarbonization in China | International  National | Emissions inventory | Full physics |
| **Vandyck et al., 2018** | Quantify the co-benefits of Nationally Determined Contributes (NDCs) from the Paris Agreement | Global | Partial equilibrium economics model | Reduced form |
| **Wolkinger et al. 2018** | Evaluate the co-benefits of low emission vehicles and active transport for three Austrian cities | International National | Transport modeling tool, Mobile emissions simulator | Lagrangian dispersion |
| **Chen et al., 2019** | Compare fourteen future energy use scenarios in India | International National | Emissions inventory | Full physics |
| **Van de Ven et al., 2019** | Assess land policies and technological subsidies on climate and air quality goals in Eastern Africa | International | Computable general equilibrium model | Reduced form |
| **Liang et al., 2019** | Compare air quality impacts of EV penetration scenarios in China | International National | Emissions inventory, Mobile emissions simulator | Full physics |
| **Lin et al., 2020** | Evaluate the air quality effects of EV adoption and charging in Taiwan | International National | Emissions inventory, Mobile emissions simulator | Full physics |

***Table S1.*** *Scope and methods of global co-benefits research. The table is color coded to specify the type of climate mitigation strategy or policy evaluated in the studies: green indicates climate policies and orange indicates transportation.*

**References**

Abel, David W., Tracey Holloway, Monica Harkey, Paul Meier, Doug Ahl, Vijay S. Limaye, and Jonathan A. Patz. 2018. “Air-Quality-Related Health Impacts from Climate Change and from Adaptation of Cooling Demand for Buildings in the Eastern United States: An Interdisciplinary Modeling Study.” *PLoS Medicine* 15 (7): 1–27. https://doi.org/10.1371/journal.pmed.1002599.

Abel, David W., Tracey Holloway, Javier Martínez-Santos, Monica Harkey, Madankui Tao, Cassandra Kubes, and Sara Hayes. 2019. “Air Quality-Related Health Benefits of Energy Efficiency in the United States.” *American Chemical Society* 53 (7): 3987–3. https://doi.org/10.1021/acs.est.8b06417.

Amann, M, I Bertok, J Borken-Kleefeld, J Cofala, C Heyes, L Hoeglund-Isaksson, Z Klimont, et al. 2009. “Greenhouse Gas and Air Pollution Interactions and Synergies (GAINS).” *Potentials and Costs for Greenhouse Gas Mitigation in Annex I Countries: Methodology, IIASA Interim Report IR-09-043*. Laxenburg, Austria.

Buonocore, Jonathan J., Patrick Luckow, Gregory Norris, John D. Spengler, Bruce Biewald, Jeremy Fisher, and Jonathan I. Levy. 2016. “Health and Climate Benefits of Different Energy-Efficiency and Renewable Energy Choices.” *Nature Climate Change* 6 (1): 100–106. https://doi.org/10.1038/nclimate2771.

Chen, Kaiyu, Hao Guo, Jianlin Hu, Sri Kota, Wenye Deng, Qi Ying, Lauri Myllyvirta, Sunil Dahiya, and Hongliang Zhang. 2019. “Projected Air Quality and Health Benefits from Future Policy Interventions in India.” *Resources, Conservation and Recycling* 142 (August 2018): 232–44. https://doi.org/10.1016/j.resconrec.2018.12.008.

Chen, Yihsu, Benjamin F. Hobbs, J. Hugh Ellis, Christian Crowley, and Frederick Joutz. 2015. “Impacts of Climate Change on Power Sector NOx Emissions: A Long-Run Analysis of the US Mid-Atlantic Region.” *Energy Policy* 84 (x): 11–21. https://doi.org/10.1016/j.enpol.2015.04.013.

Cox, William M., and Shao Hang Chu. 1996. “Assessment of Interannual Ozone Variation in Urban Areas from a Climatological Perspective.” *Atmospheric Environment* 30 (14): 2615–25. https://doi.org/10.1016/1352-2310(95)00346-0.

Dingenen, Rita Van, Frank Dentener, Monica Crippa, Joana Leitao, Elina Marmer, Shilpa Rao, Efisio Solazzo, and Luana Valentini. 2018. “TM5-FASST: A Global Atmospheric Source-Receptor Model for Rapid Impact Analysis of Emission Changes on Air Quality and Short-Lived Climate Pollutants.” *Atmospheric Chemistry and Physics* 18 (21): 16173–211. https://doi.org/10.5194/acp-18-16173-2018.

Ferrero, Enrico, Stefano Alessandrini, and Alessia Balanzino. 2016. “Impact of the Electric Vehicles on the Air Pollution from a Highway.” *Applied Energy* 169: 450–59. https://doi.org/10.1016/j.apenergy.2016.01.098.

Fiore, Arlene M., Vaishali Naik, and Eric M. Leibensperger. 2015. “Air Quality and Climate Connections Arlene.” *Journal Ofthe Air & Waste Management Association* 65 (6): 645–85. https://doi.org/10.1080/10962247.2015.1040526.

Gonzalez-Abraham, R., S. H. Chung, J. Avise, B. Lamb, E. P. Salathé, C. G. Nolte, D. Loughlin, et al. 2015. “The Effects of Global Change upon United States Air Quality.” *Atmospheric Chemistry and Physics* 15 (21): 12645–65. https://doi.org/10.5194/acp-15-12645-2015.

Grabow, Maggie L., Scott N. Spak, Tracey Holloway, Stone S. Brian, Adam C. Mednick, and Jonathan A. Patz. 2012. “Air Quality and Exercise-Related Health Benefits from Reduced Car Travel in the Midwestern United States.” *Environmental Health Perspectives* 120 (1): 68–76. https://doi.org/10.1289/ehp.1103440.

Jacob, Daniel J. 1999. *Introduction to Atmospheric Chemistry*. Princeton University Press.

Jacob, Daniel J., and Darrell A. Winner. 2009. “Effect of Climate Change on Air Quality.” *Atmospheric Environment* 43 (1): 51–63. https://doi.org/10.1016/j.atmosenv.2008.09.051.

Lelieveld, J., J. S. Evans, M. Fnais, D. Giannadaki, and A. Pozzer. 2015. “The Contribution of Outdoor Air Pollution Sources to Premature Mortality on a Global Scale.” *Nature* 525 (7569): 367–71. https://doi.org/10.1038/nature15371.

Li, Nan, Jen Ping Chen, I. Chun Tsai, Qingyang He, Szu Yu Chi, Yi Chiu Lin, and Tzung May Fu. 2016. “Potential Impacts of Electric Vehicles on Air Quality in Taiwan.” *Science of the Total Environment* 566–567: 919–28. https://doi.org/10.1016/j.scitotenv.2016.05.105.

Liang, Xinyu, Shaojun Zhang, Ye Wu, Jia Xing, Xiaoyi He, K. Max Zhang, Shuxiao Wang, and Jiming Hao. 2019. “Air Quality and Health Benefits from Fleet Electrification in China.” *Nature Sustainability* 2 (10): 962–71. https://doi.org/10.1038/s41893-019-0398-8.

Lin, Wen Yinn, Min Chuan Hsiao, Pei Chih Wu, Joshua S. Fu, Li Wei Lai, and Hsin Chih Lai. 2020. “Analysis of Air Quality and Health Co-Benefits Regarding Electric Vehicle Promotion Coupled with Power Plant Emissions.” *Journal of Cleaner Production* 247: 119152. https://doi.org/10.1016/j.jclepro.2019.119152.

Markandya, Anil, Jon Sampedro, Steven J. Smith, Rita Van Dingenen, Cristina Pizarro-Irizar, Iñaki Arto, and Mikel González-Eguino. 2018. “Health Co-Benefits from Air Pollution and Mitigation Costs of the Paris Agreement: A Modelling Study.” *The Lancet Planetary Health* 2 (3): E126–33. https://doi.org/10.1016/S2542-5196(18)30029-9.

McCollum, David L., Volker Krey, Keywan Riahi, Peter Kolp, Arnulf Grubler, Marek Makowski, and Nebojsa Nakicenovic. 2013. “Climate Policies Can Help Resolve Energy Security and Air Pollution Challenges.” *Climatic Change* 119 (2): 479–94. https://doi.org/10.1007/s10584-013-0710-y.

Meier, Paul, Tracey Holloway, Jonathan Patz, Monica Harkey, Doug Ahl, David Abel, Scott Schuetter, and Scott Hackel. 2017. “Impact of Warmer Weather on Electricity Sector Emissions Due to Building Energy Use.” *Environmental Research Letters* 12: 064014. https://doi.org/10.1088/1748-9326/aa6f64.

Peng, Wei, Junnan Yang, Xi Lu, and Denise L. Mauzerall. 2018. “Potential Co-Benefits of Electrification for Air Quality, Health, and CO2 Mitigation in 2030 China.” *Applied Energy* 218 (March): 511–19. https://doi.org/10.1016/j.apenergy.2018.02.048.

Plachinski, Steven D., Tracey Holloway, Paul J. Meier, Gregory F. Nemet, Arber Rrushaj, Jacob T. Oberman, Phillip L. Duran, and Caitlin L. Voigt. 2014. “Quantifying the Emissions and Air Quality Co-Benefits of Lower-Carbon Electricity Production.” *Atmospheric Environment* 94: 180–91. https://doi.org/10.1016/j.atmosenv.2014.03.028.

Rauner, Sebastian, Nico Bauer, Alois Dirnaichner, Rita Van Dingenen, Chris Mutel, and Gunnar Luderer. 2020. “Coal-Exit Health and Environmental Damage Reductions Outweigh Economic Impacts.” *Nature Climate Change* 10 (4): 308–12. https://doi.org/10.1038/s41558-020-0728-x.

Rauner, Sebastian, Jérôme Hilaire, David Klein, Jessica Strefler, and Gunnar Luderer. 2020. “Air Quality Co-Benefits of Ratcheting up the NDCs.” *Climatic Change*. https://doi.org/10.1007/s10584-020-02699-1.

Sampedro, Jon, Steven J. Smith, Iñaki Arto, Mikel González-Eguino, Anil Markandya, Kathleen M. Mulvaney, Cristina Pizarro-Irizar, and Rita Van Dingenen. 2020. “Health Co-Benefits and Mitigation Costs as per the Paris Agreement under Different Technological Pathways for Energy Supply.” *Environment International* 136: 105513. https://doi.org/10.1016/j.envint.2020.105513.

Shindell, Drew T., Yunha Lee, and Greg Faluvegi. 2016. “Climate and Health Impacts of US Emissions Reductions Consistent with 2 °C.” *Nature Climate Change* 6 (5): 503–7. https://doi.org/10.1038/nclimate2935.

Soret, A., M. Guevara, and J. M. Baldasano. 2014. “The Potential Impacts of Electric Vehicles on Air Quality in the Urban Areas of Barcelona and Madrid (Spain).” *Atmospheric Environment* 99 (2): 51–63. https://doi.org/10.1016/j.atmosenv.2014.09.048.

Spracklen, D. V., L. J. Mickley, J. A. Logan, R. C. Hudman, R. Yevich, M. D. Flannigan, and A. L. Westerling. 2009. “Impacts of Climate Change from 2000 to 2050 on Wildfire Activity and Carbonaceous Aerosol Concentrations in the Western United States.” *Journal of Geophysical Research* 114 (D20): 1–17. https://doi.org/10.1029/2008jd010966.

Unger, Nadine. 2012. “Global Climate Forcing by Criteria Air Pollutants.” *Annual Review of Environment and Resources* 37 (1): 1–24. https://doi.org/10.1146/annurev-environ-082310-100824.

Vandyck, Toon, Kimon Keramidas, Alban Kitous, Joseph V. Spadaro, Rita Van Dingenen, Mike Holland, and Bert Saveyn. 2018. “Air Quality Co-Benefits for Human Health and Agriculture Counterbalance Costs to Meet Paris Agreement Pledges.” *Nature Communications* 9 (1): 1–11. https://doi.org/10.1038/s41467-018-06885-9.

Vandyck, Toon, Kimon Keramidas, Stéphane Tchung-Ming, Matthias Weitzel, and Rita Van Dingenen. 2020. “Quantifying Air Quality Co-Benefits of Climate Policy across Sectors and Regions.” *Climatic Change*. https://doi.org/10.1007/s10584-020-02685-7.

Ven, Dirk Jan Van De, Jon Sampedro, Francis X. Johnson, Rob Bailis, Aikaterini Forouli, Alexandros Nikas, Sha Yu, et al. 2019. “Integrated Policy Assessment and Optimisation over Multiple Sustainable Development Goals in Eastern Africa.” *Environmental Research Letters* 14: 094001. https://doi.org/10.1088/1748-9326/ab49ad.

Weaver, C . P., X.-Z . Liang, J . Zhu, P. J . Adams, P. Amar, J . Avise, M. Caughey, et al. 2009. “A Preliminary Synthesis Of Modeled Climate Change Impacts On U.S. Regional Ozone Concentrations.” *American Meteorological Society* 90 (12): 1843–64. https://doi.org/10.1175/2009BAMS2568.1.

Wolkinger, Brigitte, Willi Haas, Gabriel Bachner, Ulli Weisz, Karl Steininger, Hans Peter Hutter, Jennifer Delcour, et al. 2018. “Evaluating Health Co-Benefits of Climate Change Mitigation in Urban Mobility.” *International Journal of Environmental Research and Public Health* 15 (5): 1–27. https://doi.org/10.3390/ijerph15050880.

Zapata, Christina, Nicholas Muller, and Michael J. Kleeman. 2013. “PM2.5 Co-Benefits of Climate Change Legislation Part 1: California’s AB 32.” *Climatic Change* 117 (1–2): 377–97. https://doi.org/10.1007/s10584-012-0545-y.

Zhao, Bin, Jiayu Xu, and Jiming Hao. 2011. “Impact of Energy Structure Adjustment on Air Quality: A Case Study in Beijing, China.” *Frontiers of Environmental Science and Engineering in China* 5 (3): 378–90. https://doi.org/10.1007/s11783-011-0357-8.
